# GlycoRNA-L and glycoRNA-S mediate human monocyte adhesion via binding to Siglec-5

**DOI:** 10.1016/j.bbamcr.2025.120017

**Published:** 2025-07-01

**Authors:** Yong Li, Yisong Qian, Evan Huang, Zain Schwarz, Hannah Tai, Katherine Tillock, Tianhua Lei, Xiaofeng Yang, Mingui Fu

**Affiliations:** a Department of Biomedical Science, School of Medicine, University of Missouri Kansas City, Kansas City, MO 64108, USA; b Department of Anesthesiology, The First Affiliated Hospital, Jiangxi Medical College, Nanchang University, Nanchang, Jiangxi 330006, China; c Key Lab for Arteriosclerology of Hunan Province, International Joint Laboratory for Arteriosclerotic Disease Research of Hunan Province, Institute of Cardiovascular Disease, University of South China, Hengyang 421001, China; d Department of Cardiovascular Sciences, Lemole Center for Integrated Lymphatics and Vascular Research, Lewis Katz School of Medicine at Temple University, Philadelphia, PA 19140, USA

**Keywords:** GlycoRNAs, Monocyte, Adhesion, Siglec-5 and endothelial cells

## Abstract

It was recently reported that RNAs can be glycosylated, *and* such glycosylated RNAs (referred to as glycoRNAs) are located on the outer cell surface. We here reported that there are two forms of glycoRNAs, named as glycoRNA-L and glycoRNA-S, robustly expressed in human monocytes. We verified that the glycoRNA-S specifically detected in human monocytes is synthesized by enzyme-catalyzed conjugation, but not artificial products of labelling probe. RNase-treatment removed both glycoRNA-L and glycoRNA-S, suggesting that they are localized on cell surface. Removing glycoRNAs significantly suppressed the interaction of human monocytes with endothelial cells, suggesting that glycoRNAs mediate human monocyte adhesion. Using flow cytometry, immunoprecipitation and northern blotting we identified Siglec-5 as the binding receptor of glycoRNAs. Siglec-5 is expressed in human endothelial cells but presented on endothelial cell surface when endothelial cells are activated. We observed that glycoRNA-L was heavily labeled with sialic acid, whereas glycoRNA-S was heavily labeled with *N*-acetylgalactosamine and *N*-acetylglucosamine. Together, these results demonstrate that two forms of glycoRNAs exist in human monocytes, which may play significant role in controlling the interaction of human monocytes and endothelial cells and contribute to the pathogenesis of inflammatory diseases.

## 1. Introduction

There are many biomolecules localized on outer cell surface including proteins, lipids and glycans, which are important to mediate cell-cell interaction and play important roles in inflammatory processes. Typically, RNAs are not expected to be present on the surface of cells. Several previous studies observed the existence of stable RNA-cell surface interactions [1,2]. The first membrane-bound RNA was discovered in bacteria [2], and it is a noncoding RNA transcribed from the same operon encoding a transmembrane protein. Following co-transcription, the noncoding RNA and the transmembrane protein assemble into a ribonucleoprotein that gets incorporated into the cell membrane [2]. Intriguingly, several recent studies identified varieties of RNA molecules localized on cellular plasma membrane. Huang et al. identified a group of RNA molecules that are stably attached to outer cell surface. All the RNA molecules they identified are RNA fragments belong to long non-coding RNAs (lncRNAs) or mRNAs [3]. Flynn et al. have reported that RNAs can be glycosylated, and such glycosylated RNA (called glycoRNAs) are located on the outer cell surfaces including human stem cells and several cancer cell lines [4]. Zhang et al. further demonstrated that the cell surface glycoRNAs are important molecules that mediate neutrophil recruitment into inflammatory areas by selective interaction with adhesion molecules on vascular endothelial cells such as P-selectin [5]. Although there are still many unclear things about the newly discovered cell surface RNAs, evidence suggests that there are small noncoding RNAs (snRNAs), which are synthesized inside cells and transported to cell plasma membrane. These intriguing discoveries open a new avenue for studying the molecular mechanisms that control inflammation and the pathogenesis of inflammatory diseases.

The pathogenesis of inflammatory diseases is not completely clear but is exacerbated by excessive infiltration of inflammatory cells including neutrophils and monocytes [6,7]. The inflammatory cells respond quickly to infection or tissue injuries by migrating from the circulation toward inflammatory sites, a process involving many cell-cell interactions. Lectin-like adhesion glycoproteins, called selectins, mediate leukocyte rolling, while the firm adhesion and subsequent transendothelial migration of leukocytes are mediated by the interaction of integrins on leukocytes with immunoglobin-like adhesion molecules ICAM-1 and VCAM-1 on endothelial cells [8–10]. However, the molecular mechanisms controlling inflammatory cell adhesion and infiltration remain incompletely understood. Targeting the process of inflammatory cell adhesion and infiltration to treat inflammatory diseases is still lacking.

Here we report that there are two forms of glycoRNAs, named as glycoRNA-L and glycoRNA-S, robustly expressed in human monocytes. Both glycoRNA-L and glycoRNA-S contributed to the adhesion of human monocytes to endothelial cells via directly binding to Siglec-5. GlycoRNA-L was heavily labeled by sialic acid, whereas glycoRNA-S was heavily labeled by *N*-acetylgalactosamine and *N*-acetylglucosamine. These studies suggest that cell surface glycoRNAs may play an important role in the regulation of inflammation and become new targets for treatment of inflammatory diseases.

## 2. Results

### 2.1. Expression of glycoRNA-L and glycoRNA-S in human monocytes

**Fig. 1.**
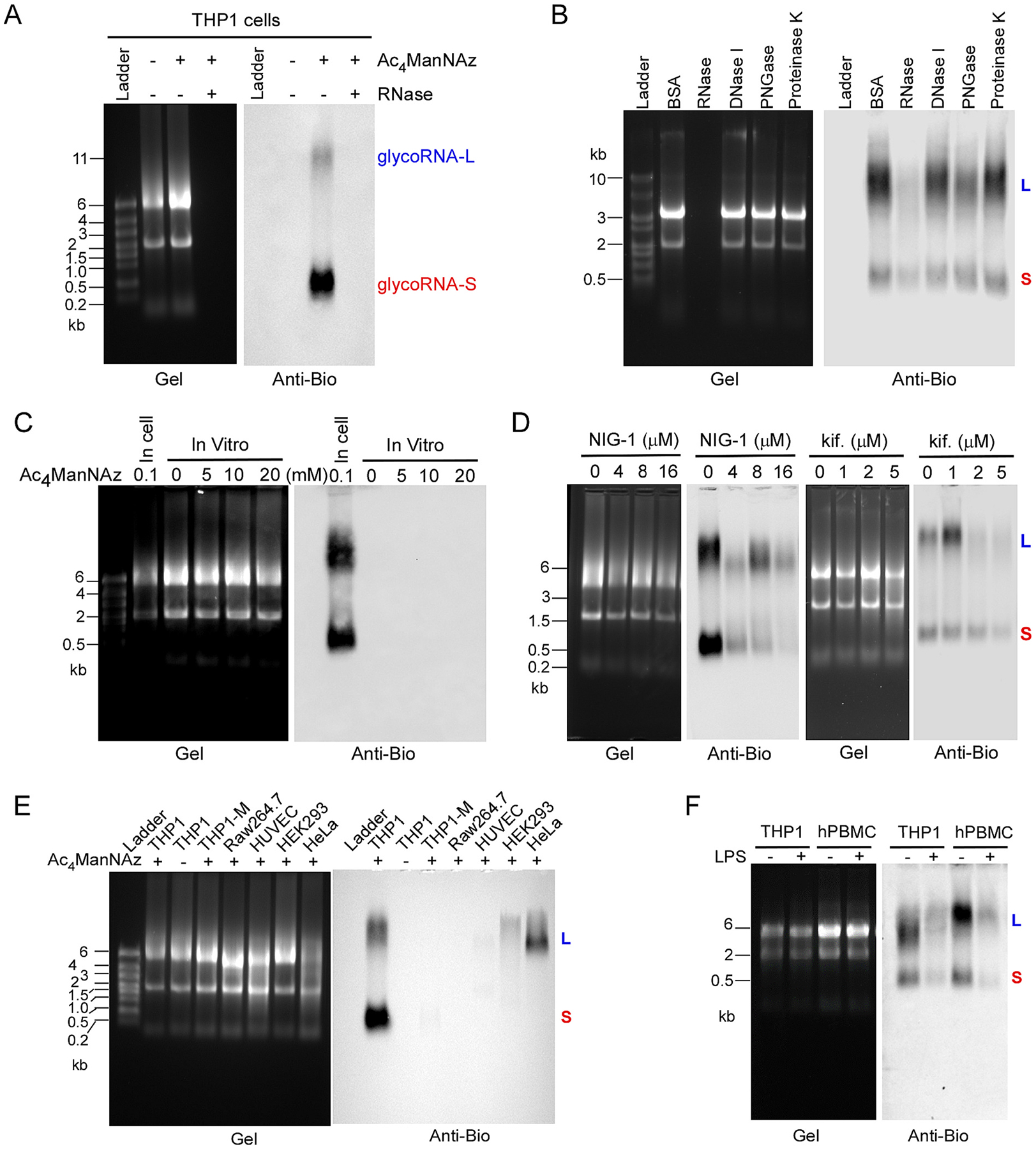
Expression of glycoRNA-L and glycoRNA-S in human monocytes. (A) THP1 cells were treated with or without Ac_4_ManNAz. RNAs were extracted from the cells and treated with or without RNase. Then the RNA samples reacted with DBCO-PEG4-biotin. RNAs were analyzed on an agarose gel (left) and then the blot was probed with an anti-biotin antibody (right). Representative images are shown. (B) 10 μg of biotinylated total RNAs from THP1 cells were incubated with 1 μl of RNase cocktail (containing RNase A 0.5 U/μ/ and RNase T 20 U/μl), DNase I (1 U/μl), PNGase F (1 U/μl) or Proteinase K (2 μg/μl) at 37 °C for 10 min. After cleaning up by zymo column, the RNA samples were analyzed by northern blot as above. (C) Blotting of cellular or in vitro Ac_4_ManNAz-labeled RNA. Cells were treated with 100 μM Ac_4_ManNAz for 24 h while native RNA in vitro was treated with 0, 5, 10 and 20 mM of Ac4ManNAz at 37 °C for 2 h. (D) THP1 cells were treated with Ac_4_ManNAz and different doses of NIG-1 or kifunensine as indicated. RNAs were extracted from the cells and analyzed by northern blot. Representative images are shown. (E) RNA from different cells treated with 100 μM Ac_4_ManNAz for 24 h and then analyzed by the same procedure as above. Representative images are shown. (F) RNA from THP1 or hPBMC (ATCC) treated with 100 μM Ac_4_ManNAz plus or not plus LPS (1 μg/ml) for 24 h and then analyzed by the same procedure as above. Representative images are shown.

To investigate whether human monocytes express glycoRNAs, we used THP1 cells, a human monocyte cell line, as a model. Following the method described by Flynn et al. [4], we metabolically labeled live THP1 cells using *N*-azidoacetylmannosamine-tetraacylated (Ac_4_ManNAz or ManNAz), which can enter the sialic acid synthesis pathway, leading to azide-modified sialic acids in glycans. Purified RNAs from labeled cells were then reacted in vitro with dibenzocyclooctyne-polyethylene-glycol-4-biotin (DBCO-PEG4-biotin) via click chemistry so that sialic-acid-containing glycoRNAs could be biotin-modified. When analyzed by denaturing electrophoresis and subsequent blotting, we observed two bands from the blot (Fig. 1A). One band was migrated with ~11 kb RNA, designated as glycoRNA-L; the other band migrated with ~0.6 kb RNA, designated as glycoRNA-S. Both glycoRNA-L and glycoRNA-S were abolished upon digestion of purified RNA by RNase prior to electrophoresis. To further verify that the two bands represent the signals from glycoRNAs, the total RNA from Ac_4_ManNAz-labeled THP1 cells was treated with RNase, DNase, PNGase and proteinase K respectively. As shown in Fig. 1B, DNase and proteinase K-treatments did not affect the glycoRNA signal, whereas treatment with an RNase cocktail (A and T1) efficiently digested the total RNA as well as the biotinylated glycoRNAs. PNGase, an enzyme that cleaves N-glycan, attenuated but not completely diminished the signals. It was recently reported that high doses of azidosugars can produce non-enzymatic protein labeling [11]. To exclude that the glycoRNA-S is an artificial form caused by labeled chemical, we incubated total RNA with 0, 5, 10 and 20 mM of Ac_4_ManNAz, which is 200 folds than the dose in cells. The RNAs were cleaned up by Zymo column and conjugated with biotin followed by northern blot analysis. As shown in Fig. 1C, in vitro incubation of total RNA with up to 20 mM Ac_4_ManNAz did not produce any signals. To further confirm that the glycoRNA-S is a new form of glycoRNAs, THP1 cells were incubated with glycan synthesis inhibitors and Ac_4_ManNAz. As shown in Fig. 1D, inhibition of oligosaccharyltransferase (OST) by NGI-1 diminished the expression of both glycoRNA-L and glycoRNA-S. Furthermore, kifunensine, an inhibitor of the N-glycan trimming enzyme α-mannosidase I, also efficiently diminished the expression of glycoRNA-L and glycoRNA-S. Together, the data above supports the presence of two forms of glycoRNAs, or more precisely sialoglycoRNAs, in human monocytes.

To explore the distribution of glycoRNA-L and glycoRNA-S in different cells, we incubated Ac_4_ManNAz with several human and mouse cell lines as indicated in Fig. 1E and followed by northern blot analysis as described above. THP1 cells expressed both glycoRNA-L and glycoRNA-S, whereas HeLa and HEK293 cells only expressed glycoRNA-L. There were no or low levels of glycoRNAs detected in THP1-derived macrophages, Raw264.7, and rest human umbilical vein endothelial cells (HUVECs). Next, we examined the expression of glycoRNA-L and glycoRNA-S in both THP1 and human primary peripheral blood mononuclear cells (hPBMC, ATCC). As shown in Fig. 1F, both glycoRNA-L and glycoRNA-S were expressed in THP1 and hPBMC. Interestingly, lipopolysaccharide (LPS)-treatment significantly decreased the expression of glycoRNA-L and glycoRNA-S in these cells.

### 2.2. GlycoRNAs mediate the interaction between human monocytes and endothelial cells

**Fig. 2.**
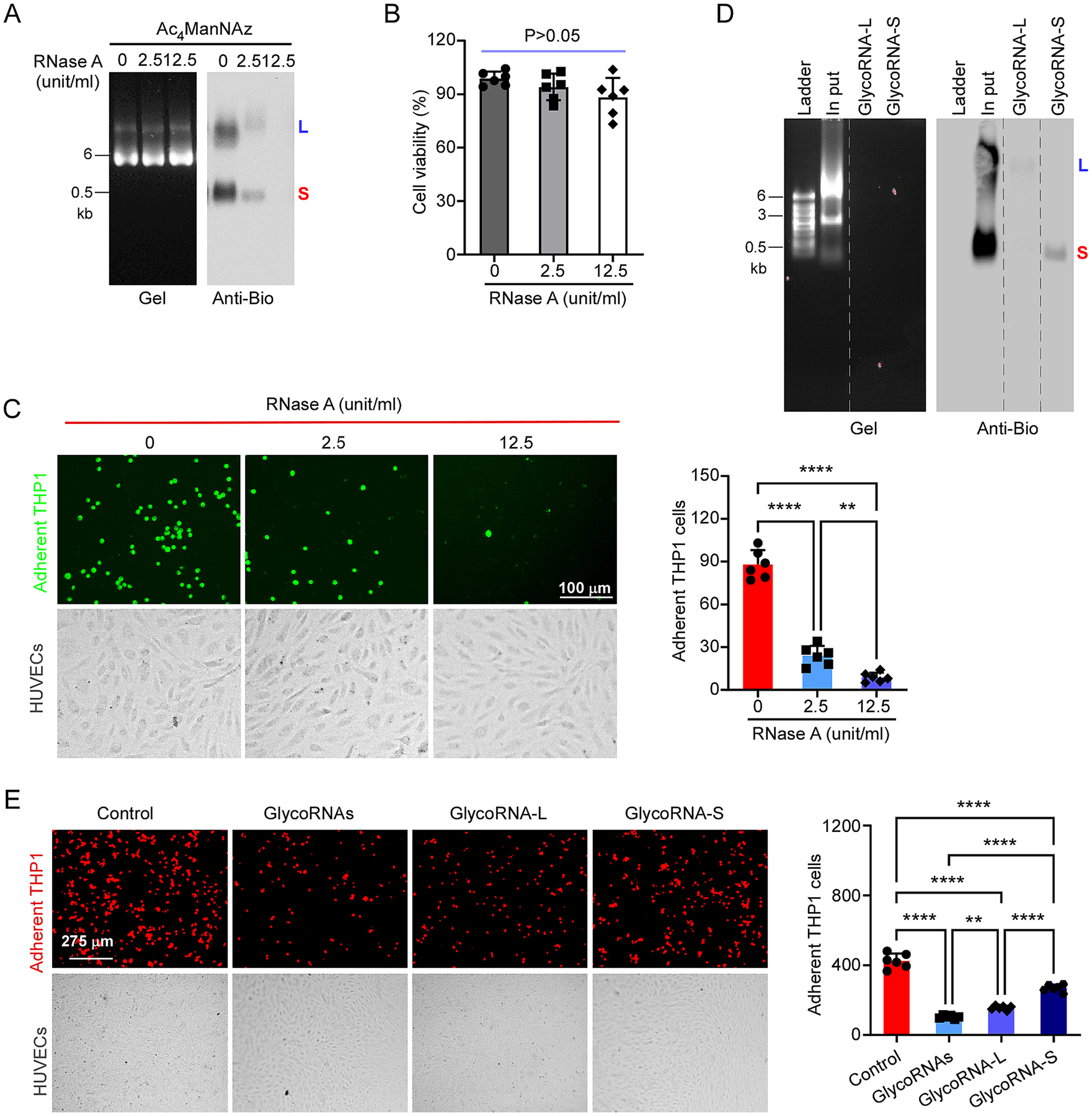
GlycoRNAs mediated human monocytes adhesion on activated endothelial cells (A) THP1 cells were metabolic labeled by Ac_4_ManNDAz for 24 h. The cells were harvested by centrifuge and washed with HBSS, then resuspended in 0.2 ml HBSS and incubated with 0, 1, or 5 μl of RNase cocktail (the concentration of RNase A is: 0, 2.5 and 12.5 units/ml). The total RNAs were isolated and analyzed as described above. (B) THP1 cells were subjected to the same treatment as RNase cocktail. The cell viability was analyzed by Trypan blue exclusion test. The data represented mean ± SD, *n* = 6, One-way ANOVA analysis followed by Tukey’s test was performed. (C) The cell images taking from RNase-treated and untreated THP1 cells then labeled by PHK67. The labeled THP1 cells incubated with activated HUVECs at 37 °C for 1 h, then the adherent THP1 cells were imaged by microscopy. The image represents one fourth of the original image to make the cells more visible (left). 6 random fields per well were imaged and the cell numbers in each field were counted by Qupath. The adherent cell numbers were averaged and presented as mean ± SD, n = 6 (right). One-way ANOVA analysis followed by Tukey’s test was performed. **P < 0.01, ***P < 0.001, ****P < 0.0001. (D) Purified glycoRNA-L and glycoRNA-S were analyzed by northern blot as described above. (E) Before adding THP1-RFP cells, the activated HUVECs were pretreated with or without 100 ng/ml of glycoRNAs, glycoRNA-L or glycoRNA-S. Then THP1-RFP cells were added into HUVECs and analyzed by the same procedure described above. The adherent cell numbers were averaged and presented as mean ± SD, n = 6 (right). One-way ANOVA analysis followed by Tukey’s test was performed. **P < 0.01, ***P < 0.001, ****P < 0.0001.

As Flynn et al. [4] reported that the glycoRNAs are localized on outer cell surface, next we examined whether RNase incubation with living cells can remove the glycoRNAs from cell surface. Briefly, the labeled THP1 cells as described above were spined down and washed once with Hank-Balanced Buffer Solution (HBSS), then 10^7^ THP1 cells were resuspended in 200 μl HBSS and incubation with 0, 2.5, 12.5 (unit/ml) of RNase A at 37 °C for 10 min. Then the cells were washed by HBSS and added 1 ml of TRIzol. The total RNAs from the cells were isolated and biotin modified as described above. As shown in Fig. 2A, the glycoRNAs can be efficiently removed by RNase treatment, which proved that glycoRNAs were expressed on cell surface. The same concentration of RNase treatment did not affect the cell viability, suggesting that RNase did not damage cell membrane and enter cells (Fig. 2B). To understand if glycoRNAs mediate monocyte adhesion on endothelial cell (EC) layer, we performed monocyte adhesion assay according to our previous publication [12]. Briefly, HUVECs were seeded on 6-well plate and stimulated with TNF-α (10 ng/ml) plus LPS (1 μg/ml) for 16h. THP1 cells were pretreated with 0, 2.5 or 12.5 (unit/ml) of RNase A in 200 μl of cells at 37 °C for 10 mins and then labeled with PHK67 (Sigma) for 5min at 37 °C before being added to HUVECs and co-cultured for 1 h. Non-adherent cells were removed by gently washing twice with cold RPMI1640 medium. The images of adherent THP1 cells were taken under microscopy. 6 images from different fields were taken from each well and the adherent cell numbers were counted by Qupath in a two-blind manner. As shown in Fig. 2C, RNase treatment significantly inhibited THP1 cell adherence on the activated endothelial cells. These data support that cell surface glycoRNAs are necessary for efficient monocyte-endothelial interactions.

To further characterize the role of glycoRNA-L and glycoRNA-S in mediating monocyte adhesion, we purified glycoRNA-L and glycoRNA-S by the procedures described by Flynn et al. [4] with some modifications. First, the biotinylated glycoRNAs were purified by Myone C1 Streptavidin beads. Then the purified glycoRNAs were separated by low-melting temperature agarose gel and the glycoRNA-L and glycoRNA-S were recovered from the gel slices. As shown in Fig. 2D, we successfully purified the glycoRNA-L and glycoRNA-S. Then we pretreated the activated HUVECs with 100 ng/ml of glycoRNAs, glycoRNA-L or glycoRNA-S followed by THP1 cell adhesion assay. As shown in Fig. 2E, pretreatment with glycoRNAs, glycoRNA-L or glycoRNA-S significantly inhibited THP1 cell adhesion, but glycoRNA-L is stronger than glycoRNA-S.

### 2.3. Identification of Siglec-5 as the binding partner of glycoRNAs from THP1 cells

**Fig. 3.**
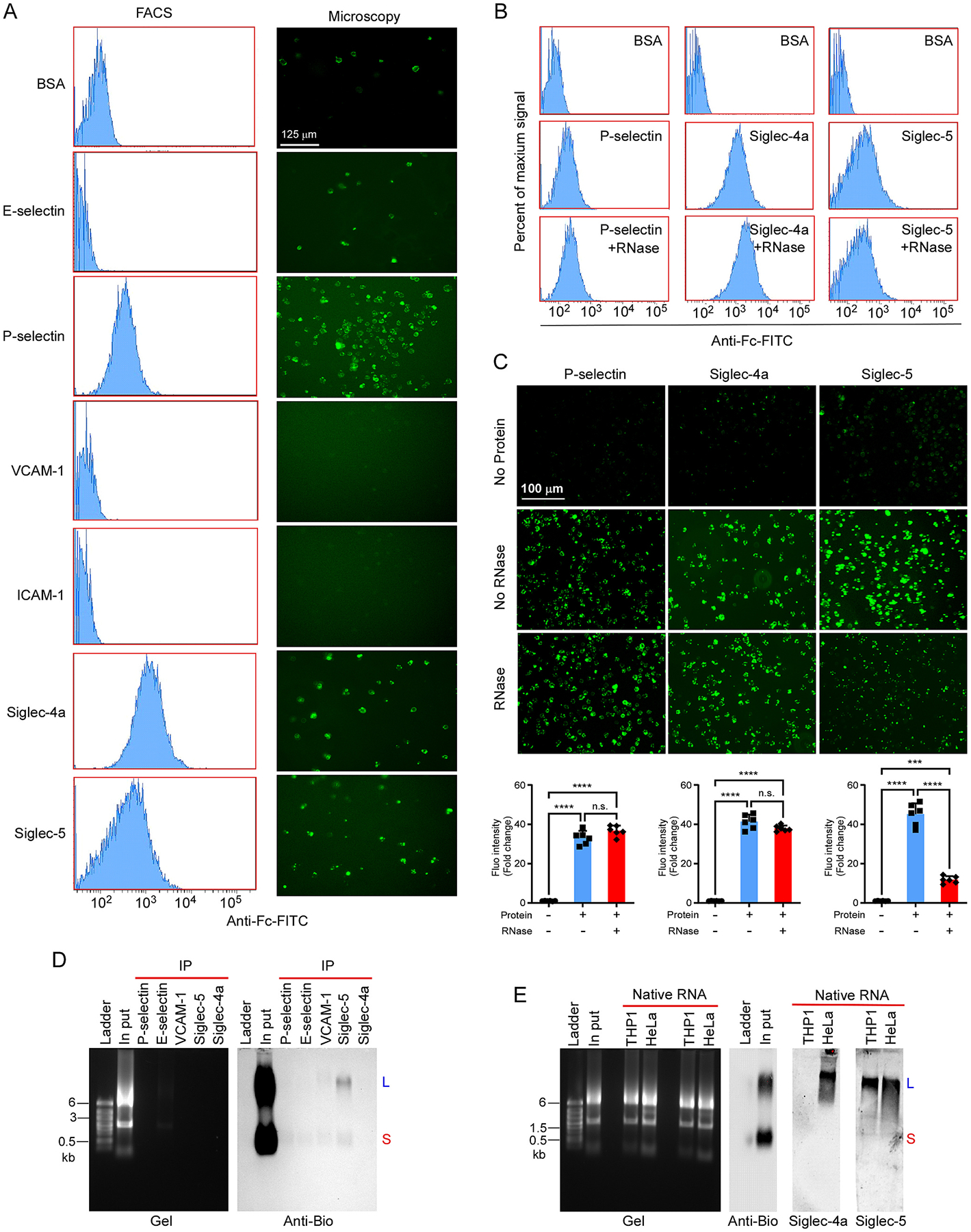
Identification of Siglec-5 as the binding partner of glycoRNAs from THP1 cells. (A) FACS analysis of THP1 cells labeled by pre-complex of *E*-selectin-Fc, P-selectin-Fc, VCAM-1-Fc, ICAM-1-Fc, Siglec-4a-Fc, Siglec-5-Fc and anti-Fc-FITC (left). The images taken from these cells by microscopy (right). (B) FACS analysis of THP1 cells pretreated with or without RNase then stained with the indicated pre-complex. (C) The images taken from these cells in (B) by microscopy. The fluorescence intensity from biological triplicate was analyzed by Cytation 3 Cell Imaging Multi-mode Reader (Biotek Instruments) and presented as mean ± SD, *n* = 6. One-way ANOVA was performed. **P < 0.01, ***P < 0.001, ****P < 0.0001, n.s means no significance. (D) Biotinylated RNA was incubated with pre-binding anti-Fc-beads with indicated protein-Fc chimera and then analyzed by northern blot as described above. (E) Native RNAs were analyzed by northern blot. After blocking, the membrane was probed by pre-complex of Siglec-4a-Fc (1.5 μg/ml) or Siglec-5-Fc (1.5 μg/ml) with anti-Fc-HRP (1.5 μg/ml) for 2 h at 4 °C. The blot was imaged by iBright 1500. Blot of biotinylated RNA from labeled THP1 cells was probed by anti-biotin-HRP.

Next, we searched for the target proteins on HUVECs that might bind to the glycoRNAs from THP1 cells. First, we purchased the commercially available endothelial adhesion proteins including P-selectin, *E*-selectin, VCAM-1, ICAM-1, Siglec-4a and Siglec-5-Fc chimeras (Biolegend). The first four proteins are reported adhesion molecules of endothelial cells. Siglec-5 was recently reported to be expressed in endothelial cells [13]. Siglec-4a (myelin-associated glycoprotein, MAG) was expressed in center nerves system [14] and included as a control. Then we probed their binding to THP1 cells by flow cytometry and microscopy. As shown in Fig. 3A, among of 6 recombinant proteins tested, only P-selectin, Siglec-4a and Siglec-5 strongly bind to THP1 cells. Then, we pretreated THP1 cells with RNase before incubation with P-selectin, Siglec-4a or Siglec-5-Fc followed by the analysis of flow cytometry and microscopy. As shown in Fig. 3B&C, only Siglec-5 binding was diminished by RNase treatment. To further confirm that Siglec-5 directly binds to the glycoRNAs from THP1 cells, we performed an immunoprecipitation experiment. Briefly, biotinylated total RNAs from Ac_4_ManNAz-labeled THP1 cells were incubated with P-selectin, *E*-selectin, VCAM-1, Siglec-4a and Siglec-5-Fc chimeras and then pulled down by incubation with anti-Fc agarose beads. The bound biotinylated glycoRNAs were detected by northern blot analysis as described previously. As shown in Fig. 3D, only Siglec-5 can efficiently pulled down glycoRNA-L and glycoRNA-S. Next, we directly probed the northern blot membrane with P-selectin, *E*-selectin, Siglec-4a and Siglec-5-Fc chimera and anti-human Fc-HRP complex as described by Zhang et al. [5]. As shown in Fig. 3E, only Siglec-5 specifically binds to the native glycoRNAs from THP1 cells. Both Siglec-4a and Siglec-5 can bind to the native glycoRNAs from HeLa cells. Interestedly, Siglec-5 preferentially binds to glycoRNA-L but not glycoRNA-S. P-selectin and E-selectin did not bind to the glycoRNAs from THP1 and HeLa cells (data not shown).

### 2.4. Siglec-5 is required for the interaction of human monocyte and endothelial cells

**Fig. 4.**
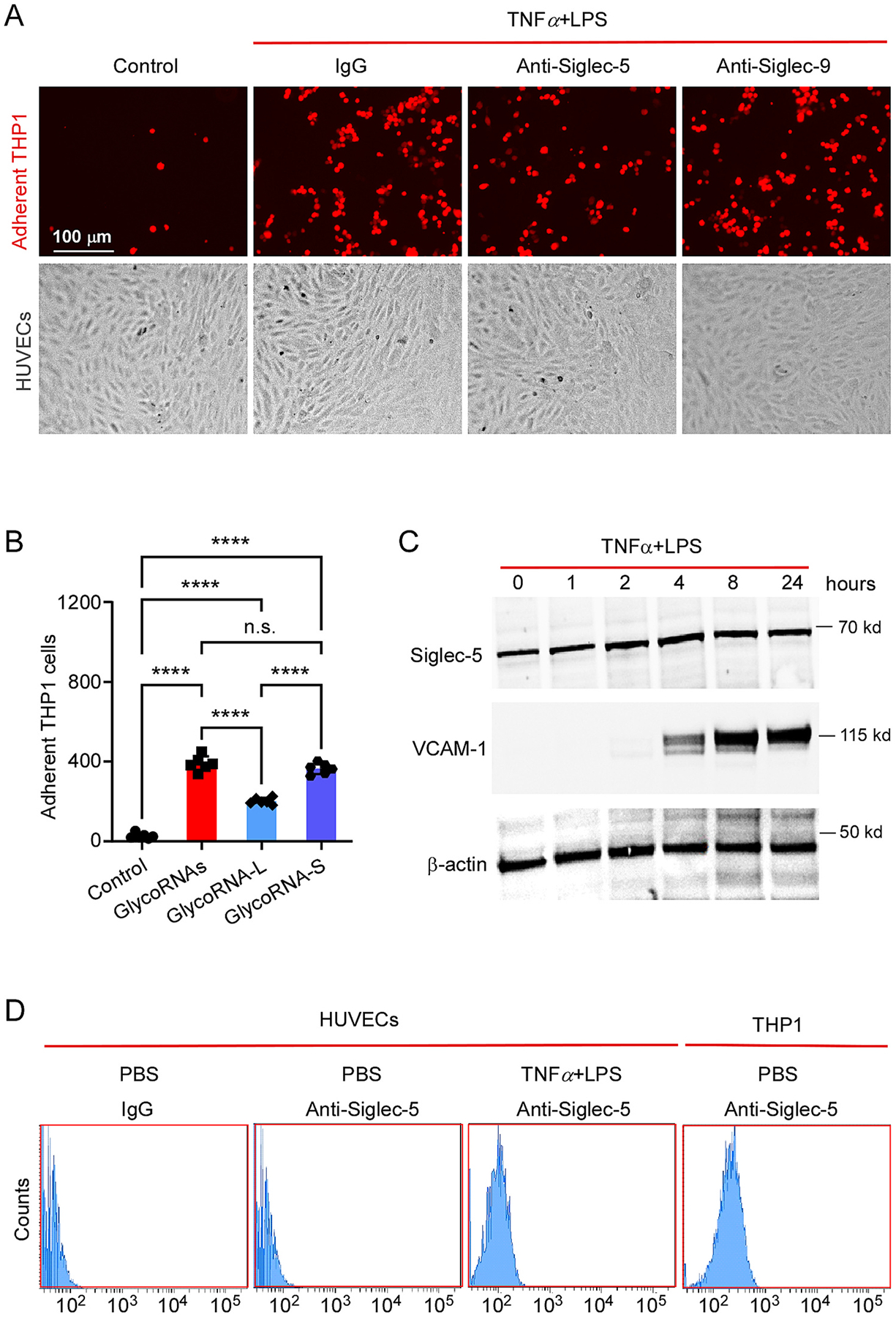
Siglec-5 is required for mediating the interaction of human monocytes and endothelial cells. (A) Before adding THP1-RFP cells, the activated HUVECs were pretreated with or without 1.5 μg/ml of anti-siglec-4a or anti-Siglec-5. Then THP1-RFP cell adherent was analyzed by the same procedure described above. (B) 6 random fields per well were imaged from (A) and the cell numbers in each field were counted by Qupath. The adherent cell numbers were averaged and presented as mean ± SD, n = 6. One-way ANOVA analysis followed by Tukey’s test was performed. **P < 0.01, ***P < 0.001, ****P < 0.0001. (C) Western blot of cell lysates from HUVECs treated with TNFα (10 ng/ml) and LPS (1 μg/ml) for different time points as indicated. The blot was probed by primary antibodies as indicated and imaged by secondary antibody-HRP. (D) HUVECs were stimulated with TNFα and LPS overnight and then incubated with anti-Siglec-5 and Alex468-tagged anti-IgG secondary antibody complex. THP1 cells were also stained as a control. The stained cells were analyzed by Flow cytometry.

Next, we tested whether Siglec-5 is required for the interaction of human monocyte and endothelial cells. Before incubation with THP1 cells, the activated HUVECs were pretreated with 1.5 μg/ml of anti-Siglec-5 or anti-Siglec-9. As shown in Fig. 4A&B, treatment with anti-Siglec-5 but not anti-Siglec-9 significantly attenuated the THP1 cell adhesion. These results suggest that Siglec-5 is a binding partner of glycoRNAs from THP1 cell, which is required for efficient interaction of human monocyte and endothelial cells. To examine the expression of Siglec-5 in HUVECs, HUVECs were treated with TNFα and LPS for different times as indicated in Fig. 4C. The cell lysates were subjected to western blot analysis with the antibodies as indicated. As shown in Fig. 4C, VCAM-1 was significantly induced by TNFα and LPS treatment as expected. However, Siglec-5 was constitutively expressed in rest HUVECs and slightly increased (~2 folds) after stimulation with TNFα and LPS. To further examine if Siglec-5 is present on the cell surface of HUVECs, HUVECs were stimulated with TNFα and LPS overnight and then incubated with anti-Siglec-5 and Alex468-tagged anti-IgG secondary antibody complex. THP1 cells were also stained as a control. The stained cells were analyzed by Flow cytometry. As shown in Fig. 4D, Siglec-5 is present on the cell surface of activated HUVECs and THP1 cells but not on rest HUVECs.

### 2.5. Characterization of the structures of glycoRNA-L and glycoRNA-S

**Fig. 5.**
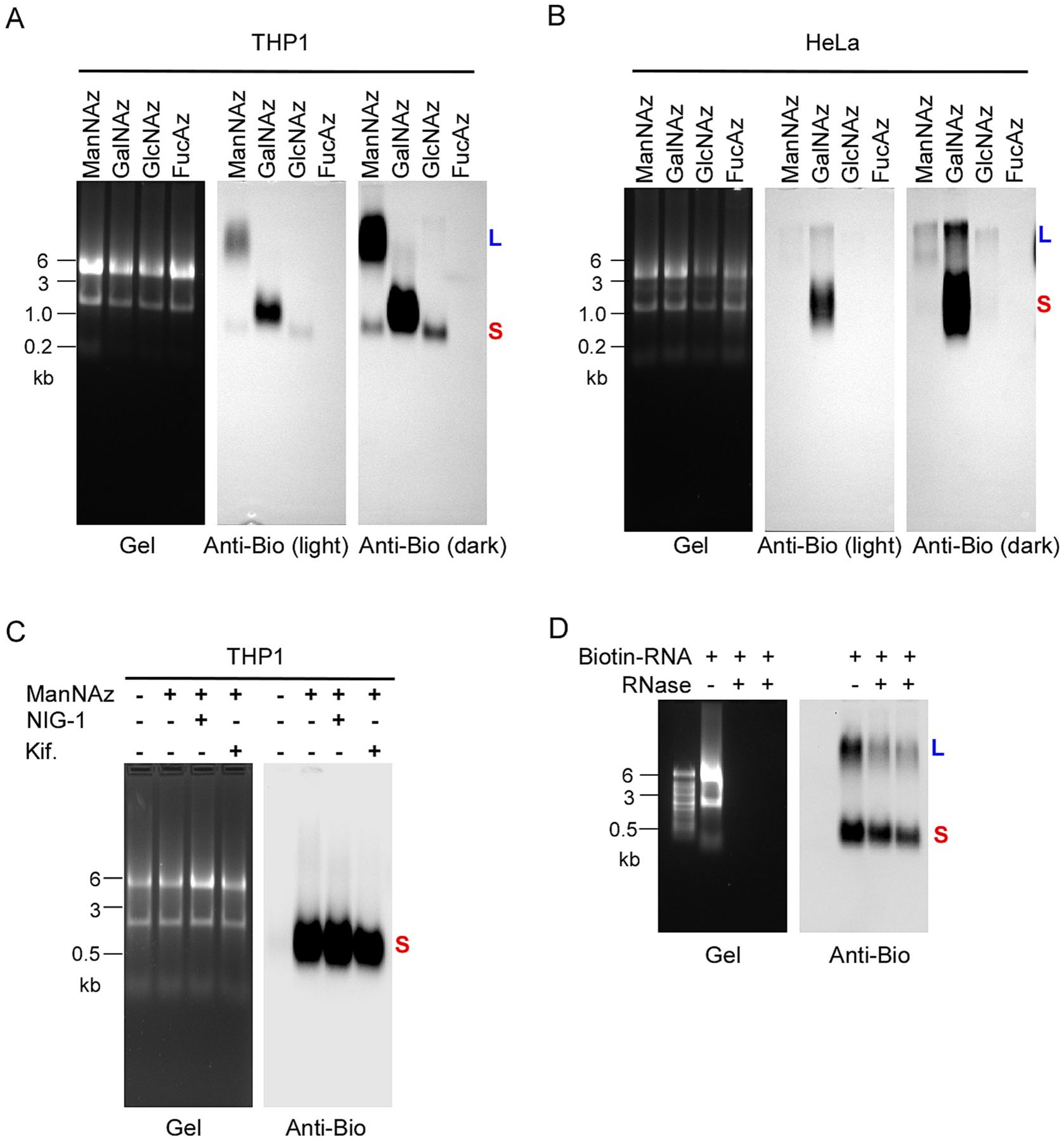
Characterization of structures of glycoRNA-L and glycoRNA-S in THP1 cells. (A) THP1 cells were labeled by 100 μM of Ac_4_ManNAz (ManNAz), GalNAz, GlcNAz and FucAz for 24 h respectively. RNAs were extracted from the cells. Then the RNA samples reacted with DBCO-PEG4-biotin. RNAs were analyzed on an agarose gel (left) and then blotted with an anti-biotin antibody (right). Representative images are shown. (B) Blot of RNA from HeLa cells labeled by same reagents as above. (C) THP1 cells were pretreated with or without NIG-1 (10 μM/L) or kifunensine (5 μM/L) for 1 h and then incubated with GalNAz (100 μM/L) for 24 h. The RNA was isolated and analyzed as described above. (D) 10 μg biotinylated total RNA from Ac_4_ManNAz-labeled THP1 cells were treated with 1 μl RNase cocktail and then directly loaded on the denaturing gel and analyzed by same procedure as above.

Next, we try to characterize the structures of glycoRNA-L and glycoRNA-S. As previous report suggested, glycoRNAs are synthesized in vivo by conjugating the glycan chains with the fragments from small non-coding RNAs [4]. A recent report demonstrated that glycan chain is attached to the RNA through covalently binding 3-(3-amino-3-carboxypropyl)uridine (acp^3^U, ref. 15). In mammalian, the glycan chains on glycoproteins and glycolipids are commonly composed of sialic acid, galactose, glucose, *N*-acetylgalactosamine (GalNAc), *N*-acetylglucosamine (GlcNAc), mannose, fucose and xylose [16]. To determine the composition of glycan chains of glycoRNA-L and glycoRNA-S, we purchased four commercially available azido-sugar molecules Ac_4_ManNAz (ManNAz), *N*-azidoacetylgalactosamine-tetraacylated (GalNAz), *N*-azidoacetylglucosamine-tetraacylated (GlcNAz) and 9-azido-fucose (FucAz, ref. 17). We labeled the THP1 and HeLa cells with these probes and analyzed by northern blot as described above. As shown in Fig. 5A, in THP1 cells, glycoRNA-L was heavily labeled by ManNAz, whereas glycoRNA-S was heavily labeled by GalNAz and GlcNAz. As GalNAz can interchange into GlcNAz by catalyzing with GALE. Both GalNAz and GlcNAz are direct materials for glycan chain synthesis. Feeding cells with GalNAz, the entity we detected may not only contain GalNAz but also contain GlcNAz. Similarly, feeding cells with GlcNAz, the bands we detected may not only contain GlcNAz, but also contain GlaNAz. These results suggest that the compositions of glycan chains of glycoRNA-L and glycoRNA-S are various. At least, glycoRNA-L has more sialic acid than glycoRNA-S, whereas glycoRNA-S has more GalNAc and GlcNAc than glycoRNA-L. In HeLa cells, glycoRNA-L was labeled by Ac_4_ManNAz, GalNAz and GlcNAz. GlycoRNA-S is heavily labeled by GalNAz (Fig. 5B). Taking together, these results suggest that the glycan compositions between glycoRNA-L and glycoRNA-S are vary, which may make their function also different. In addition, in different cells, the glycan compositions of glycoRNAs are also different, which may make them have specific function. Interestingly, we further observed that the GalNAz-labeled glycoRNA-S was not diminished by NIG-1 and kifunensine, suggesting that the GalNAz-labeled glycan is not N-glycan (Fig. 5C). Zhang et al. recently reported that after PNGase F digest, the RNA species in glycoRNAs were migrated as a single band around 40 bp [5]. We reason that the RNA portions on glycoRNA-L and glycoRNA-S may be similar. As shown in Fig. 5D, removing the RNA portion by RNase only slightly affects the migration of both glycoRNA-L and glycoRNA-S, which suggests that the migration difference between glycoRNA-L and glycoRNA-S is majorly determined by their glycan chains but not RNA fragments. Further decoding the structures of glycoRNA-L and glycoRNA-S is critical for understanding their functional significance.

## 3. Discussion

In this study, we demonstrate that there are two forms of glycoRNA, or more precisely sialoglyRNAs, robustly expressed in human monocytes. The glycoRNA-L migrated with RNA around 11 kb and glycoRNA-S migrated with RNA around 0.6 kb. These two forms of glycoRNAs also appeared on the blots in several previous reports [4,15,18]. As the glycoRNA-S is much weaker in their blots, it was thought of as a non-specific band catalyzed by labelling chemicals. In our experiments, incubation of total RNAs with 200 folds of concentration of labelling Ac_4_ManNAz did not generate the small band. Treatment of cells with NIG-1 and kifunensine abolished both glycoRNA-L and glycoRNA-S, which further demonstrated that glycoRNA-S is a new form of glycoRNAs, which is produced by enzyme-catalyzed procedures.

The expression of glycoRNA-L is broader than glycoRNA-S. After reviewing all of reports published so far [4,5,15–19], there are 16 different cell lines expressed glycoRNA-L including H9, 4188, HeLa, CHO, HOXB8, mouse bone marrow-derived neutrophils, THP1, HEK293, U2OS, human primary alveolar epithelial cells (hPAEpC), HUH7, HAP1, human primary T cells, B cells, monocytes and neutrophils. However, there are only 4 cell lines expressed glycoRNA-S including THP1, 4188, GM78, and hPBMC. As the current detection of glycoRNAs was using Ac_4_ManNAz, which specifically labels sialic acid. There may be other forms of glycoRNAs expressed in cells, which do not contain sialic acid. For instance, a recent report showed that there are more O-glycan-conjugated glycoRNAs exist in mammalian cells if detected by rPAL [20].

The interaction of leukocytes with endothelial cells is the first step of inflammatory response during infection or tissue injury. Glycan-conjugated molecules usually mediate leukocyte adhesion by recognizing their receptors on endothelial cells [21]. Our results demonstrate that both glycoRNA-L and glycoRNA-S are involved in mediating the interaction of human monocytes and endothelial cells. We identified Siglec-5 as the receptor for glycoRNAs from THP1 cells. Siglec-5 is a CD33-related Siglecs. It is highly expressed in neutrophils, monocytes and B cells [22]. It prefers to bind to sialic acid-containing glycan-conjugated molecules [23]. As glycoRNA-L is heavily labeled with sialic acid, it is not surprising that Siglec-5 strongly binds to glycoRNA-L, but not glycoRNA-S. The functional studies also confirmed that glycoRNA-L plays more important role than glycoRNA-S in mediating human monocyte adhesion. Western blot showed that Siglec-5 was constitutively expressed in HUVECs and slightly increased (~2 folds) during EC activation. However, Flow cytometry analysis suggests that Siglec-5 is not present on cell surface at rest condition, whereas it may be present on cell surface when ECs are activated. These results may explain why human monocytes only adherent to activated ECS but not rest ECs.

As Zhang et al. reported that the glycan portion but not RNA portion, mediates cell interaction [5], resolving the composition and structure of glycan chains of glycoRNAs is very important to understand their function. Our results suggest that the sugar compositions in the glycan chains of glycoRNA-L and glycoRNA-S may be distinguished. At least, glycoRNA-L is heavily labeled with sialic acid, whereas glycoRNA-S is heavily labeled by GalNAc and GlcNAc. Their glycan structures may determine their functional significance. In addition, as Zhang et al. observed that after removing glycan chains by PNGase, the RNA species are migrated as a single band around 40 bp. In our experiment, removing RNA portion by RNase only make the bands for both glycoRNA-L and glycoRNA-S migrate slightly faster, further confirming that the migration nature of glycoRNA-L and glycoRNA-S is due to the glycan chains but not the RNA portion.

There may be many more different types of RNA molecules localized on cell surface. Some RNA molecules are modified by glycans, the others may be modified by other molecules. For example, Huang et al. identified a group of RNA molecules on cell surface, which is mainly composed of RNA fragments from long non-coding RNAs and mRNAs [3]. At least two groups generated RNA libraries from lectin-purified glycoRNAs did not contain those RNA species [4,5], suggesting that those RNA molecules may be modified by other conjugates but not glycans. Identification of the whole cell membrane RNA family would be important to understand their physiological function and pathological involvement.

In summary, here we report the identification of two glycoRNA forms in human monocytes. By serial experiments, we investigated their expression, function, and mechanisms. The limitation of these studies is obvious. First, in Vivo the functional significance of the two forms of glycoRNAs is not addressed. Second, the structure of glycoRNA-L and glycoRNA-S was not fully elucidated. More studies are needed to further characterize their functions and structures.

## 4. Methods

### 4.1. Cell culture

All cells were grown at 37 °C and 5 % CO_2_. HeLa, HEK293 and Raw264.7 cells (all from ATCC) were cultured in 1 × DMEM based media (ThermoFisher Scientific) supplemented with 10 % heat inactivated fetal bovine serum (FBS, ThermoFisher Scientific) and 1 % penicillin/streptomycin (P/S, ThermoFisher Scientific, *V*/V). THP1 and THP1-RFP cells (gifts from Dr. Daping Fan) were cultured in RPMI-1640 based media (ThermoFisher Scientific) with 1 mM HEPES, 1 mM Sodium Pyruvate, 0.001 % β-mercaptoethanol and glutamine supplemented with 10 % FBS. HUVECs (ThermoFisher Scientific) were cultured in EBM2 growth media (Lonza). THP1-derived macrophages were from THP1 cells induced by PMA (Sigma) 200 ng/ml for 5 days and cultured in the same medium as THP1 cells.

### 4.2. Metabolic labeling of the cells

Stocks of *N*-azidoacetylmannosamine-tetraacylated (Ac_4_ManNAz or ManNAz, Tocris Bioscience, Cat#:7479) were made to 500 mM in sterile dimethyl sulfoxide (DMSO). In cell experiments Ac_4_ManNAz was used at a final concentration of 100 μM. In vitro experiments with Ac_4_ManNAz used 0, 5, 10, or 20 mM Ac_4_ManNAz (up to 200× the in-cell concentrations) for 2 h at 37 °C. Working stocks of glycan-biosynthesis inhibitors were all made in DMSO at the following concentrations and stored at −80 °C: 10 mM NGI-1 (Tocris Bioscience, Cat#:6652), 10 mM Kifunensine (Tocris Bioscience, Cat#:3207), All compounds were used on cells for 24 h and added simultaneously with Ac_4_ManNAz for labeling. N-azidoacetygalactosamine-tetraacylated (GalNAz, Thermo Fisher Scientific, Cat#:88905), *N*-azidoacetylglucosamine-tetraacylated (GlcNAz, Thermo Fisher Scientific, Cat#:88903) and 9-azido-fucose (FucAz, Synthase, Cat#:AF415) were all made to 500 mM stock and used at a final concentration of 100 μM.

### 4.3. RNA extraction and purification strategies

For total RNA isolation, TRIzol reagent (Thermo Fisher Scientific, Cat#15596018) was always used as a first step to lyse and denature cells or tissues. After homogenization in TRIzol by pipetting, samples were incubated at 37 °C for 10 mins to further denature non-covalent interactions. Phase separation was initiated by adding 0.2 × volumes of 100 % chloroform, vertexing to mix well, and finally spinning down at 12,000 ×g for 15 min at 4 °C. The aqueous phase was carefully removed, transferred to a fresh tube and mixed with equal volume of isopropanol. The mixture was put in 4 °C for 10 mins and then centrifuged at 12,000 ×*g* for 10 min at 4 °C. The RNA pallet was washed by 1 ml of 75 % ethanol (EtOH). The RNA pellet was dry at room temperature and dissolved by RNase-free water. After enzymatic treatment or biotin-conjugating, the samples were always purified by Zymo column (Research Products Int., Cat#:ZR1017).

### 4.4. Enzymatic treatment of RNA samples and cells

To digest RNA the following was used: 1 μL of RNase cocktail (0.5 U/μL RNaseA and 20 U/μL RNase T1, ThermoFisher Scientific, Cat#: AM2286), DNase I (Zymo Research, Cat#:E1009), Proteinase K (ThermoFisher Scientific, Cat#:100005393) or PNGase F (NEB, Cat#:P0705) was added into 20 μl of total RNA at 37 °C for 10 mins. The reaction mixture was purified by Zymo column. To digest biotinylated RNA, 1 μL of RNase cocktail was added into 20 μl of biotinylated RNA 37 °C for 10 mins. The reaction mixture was directly mixed with equal volume of dfGLB II (95 % Formamide, 18 mM EDTA, and 0.025 % SDS) for northern blot analysis. To digest the RNA on the cells, 10^7^ cells were washed once by HBSS and resuspended by 0.2 ml of HBSS, then added with 0, 1 or 5 μl of RNase cocktail at 37 °C for 10 mins.

### 4.5. Copper-free click conjugation to RNA

Copper-free conditions were used in all experiments to avoid copper in solutions during the conjugate of biotin to the azido sugars. All experiments used dibenzocyclooctyne-PEG4-biotin (DBCO-PEG4-biotin, Tocris Bioscience, Cat#:7480) as the alkyne half of the cycloaddition. To perform the SPAAC, RNA in pure water was mixed with 1× volumes of “dye-free” Gel Loading Buffer II (df-GLBII, 95 % Formamide, 18 mM EDTA, and 0.025 % SDS) and 500 μM DBCO-biotin. Typically, these reactions were 30 μL df-GLBII, 27 μL RNA, 3 μL 10 mM stock of the DBCO reagent. Samples were conjugated at 55 °C for 10 min to denature the RNA and any other possible contaminants. Reactions were stopped by adding 2× volumes (120 μL) of RNA Binding Buffer (Zymo), vertexing, and finally adding 3× volumes (180 μL) of 100 % EtOH and vertexing. This binding reaction was purified over the Zymo column as instructed by the manufacturer and analyzed by gel electrophoresis as described below.

### 4.6. RNA gel electrophoresis, blotting, and imaging

Blotting analysis of Ac_4_ManNAz-labeled RNA was performed according to the procedures described by Flynn et al. with the following modifications. RNA on the column was eluted by 18 μl H_2_O and then added 18 μl of df-GLBII with ethidium bromide (EB, ThermoFisher Scientific). To denature, RNA was incubated at 55 °C for 10 min and crashed on ice for 3 min. Samples were then loaded into a 1 % agarose-formaldehyde denaturing gel (NorthernMax Kit, ThermoFisher Scientific) and electrophoresed at 60 V for 60 min. Total RNA was then visualized in the gel using iBright 1500 (Invitrogen). RNA transfer to nitrocellulose membrane (NC, 0.45 μm, ThermoFisher Scientific) occurred as per the NorthernMax protocol for 2–16 h at 25 °C. After transferring, RNA was crosslinked to the NC using UV-C light (0.18 J/cm^2^). NC membranes were then blocked with Protein Free Blocking Buffer, PBS (Li-Cor Biosciences, Cat#:927–90,001) for 60 min at 25 °C. After blocking, the blot was incubated with anti-Biotin-HRP (1:1000, ThermoFisher Scientific, Cat#:50195911) for 2–16 h at 4 °C. Excess anti-biotin-HRP was briefly washed from the membranes by 0.1 % Tween-20 (Sigma) in 1× PBS. NC membranes were imaged on iBright 1500 (Invitrogen).

For detecting the interaction of recombinant protein-Fc chimera with glycoRNAs, native RNA extracted from unlabeled cells were subjected to gel electrophoresis and blotting as described above. Recombinant protein-Fc (1.5 μg/ml, BioLegend) was pre-complexed with anti-Fc-HRP (1.5 μg/ml, ThermoFisher Scientific, Cat#:A18817) in 5 ml FACS buffer at 4 °C for 1 h. After blocking, the NC membrane was incubated with the pre-complexed protein and second antibody solution at 4 °C for 2 h. Excess anti-Fc-HRP was briefly washed from the membranes by 0.1 % Tween-20 (Sigma) in 1× PBS. NC membranes were imaged on iBright 1500 (Invitrogen).

### 4.7. Analysis of monocyte adhesion on ECs

To perform the adhesion assay, HUVECs were seeded on 6-well plates (Falcon). When HUVECs formed a dense layer and completely covered the dish, a final concentration of 10 ng/mL TNF-α and 1 μg/ml of LPS (Sigma) were added to the medium and the HUVECs were cultured for an additional 16–24 h. 10^7^ THP1 cells or THP1-RFP cells were spin down and washed once by HBSS with Ca^2+^ and Mg^2+^ (Gibco). THP1 or THP1-RFP cells were then incubated with 0.2 mL HBSS with Ca^2+^ and Mg^2+^ supplemented with 0, 1 or 5 μl of RNase cocktail for at 37 °C for 10 min. THP1 cells were labeled by 1 μM PHK67 (Sigma) according to the manufacturer’s instruction. 10^6^ Labeled THP1 cells or THP-1-RFP cells were added into each well and incubated for 1 h. Unattached or loosely attached THP1 cells were washed twice by 2 ml HBSS with Ca^2+^ and Mg^2+^. Attached THP1 cells were fixed by adding 4 % formaldehyde for 15 mins at room temperature. The cells then were imaged by microscopy (Evos FL Auto 2, Invitrogen). At least 6 images were taken from each well and the adhesion cells were counted by Qupath. For blocking experiments with anti-Siglec-5 or anti-Siglec-9, HUVECs were pre-treated with or without 10 ng/mL TNF-α and 1 μg/ml of LPS (Sigma) for 16–24 h. The cells were then incubated with 1.5 μg/ml of IgG (Control), anti-Siglec-5 (R&D Systems, Cat#:MAB10721) or anti-Siglec-9 (R&D Systems, Cat#:MAB1139) for 1 h. After washing once, 10^6^ THP-1-RFP cells were added into each well and incubated for 1 h. Unattached or loosely attached THP1-RFP cells were washed twice by 2 ml HBSS with Ca^2+^ and Mg^2+^. Attached THP1-RFP cells were then analyzed as described as above.

### 4.8. Fluorescence-activated cell sorting (FACS) and microscopy analysis

THP1 cells were grown as described above and spin down by centrifuge. Cells were resuspended in FACS Buffer (0.5 % bovine serum albumin, ThermoFisher Scientific) in 1× PBS, counted, and aliquoted to 1,000,000 cells per 200 μL FACS Buffer, incubating on ice for 30 min to blocking. For RNase digestions, 1 μl of RNase cocktail was added to the blocking buffer and incubated at 37 °C for 10 min and then put on ice for 20 min. After blocking, cells were brought to 25 °C for 5 min, then spun for 5 min at 4 °C and 350 × g. Cells were washed once with 150 μL FACS Buffer and spun as above. 1.5 μg/ml recombinant protein-Fc chimera was precomplexed with 1.5 μg/ml anti-human Fc-FITC (Sigma, Cat#: F9512) in 200 μl FACS buffer on ice at dark for 1 h. Then the THP1 cells were resuspended with 200 μl of the pre-complexed recombinant protein-Fc-Secondary solution and incubated on ice in the dark for 30 min, washed once with 200 μl FACS buffer and resuspended in 200 μl FACS buffer. 150 μl of cells were sent to our FACS core for analysis and the other 50 μl of cells was fixed by adding 50 μl of 4 % formaldehyde for 15 min at room temperature. The fixed cells were spread onto 12 well plates and centrifuged for 5 min. Then the cells were imaged by microscopy (Evos FL Auto 2, Invitrogen).

### 4.9. Immunoprecipitation

50 μL anti-Fc beads (Invitrogen, Cat#:80108G) were pro-conjugated with 10 μg of recombinant protein-Fc chimera in samples buffer (50 mM Tris, pH 7.4, 150 mM NaCI, 0.5 % Triton X-100) for 2 h at 4 °C. After washing three times with samples buffer, beads were incubated with 10 μg of biotinylated RNA from Ac_4_ManNAz-labeled THP1 cells for 2 h at 4 °C. After washing three times with samples buffer, the beads were suspended in 50 μL RNA binding buffer and heated for 5 min at 50 °C. Remove the beads and transfer the RNA extract solution to a new tube. Here, 100 μL of pure water was added and vortexed for 10 s, and then 300 μL of 100 % ethanol was added and vortexed for 10 s. The RNAs were purified over a Zymo column followed by northern blot analysis as described above.

### 4.10. Purification of glycoRNA-L and glycoRNA-S

The purification of biotin-labeled glycoRNAs was achieved by streptavidin beads with the following steps: 20 μL of MyOne C1 Streptavidin beads (Thermo Fisher Scientific, Cat#:65001) per reaction were blocked with 50 ng/μL glycogen (Thermo Fisher Scientific, Cat#:R0561) and 1 U/μL RNase Inhibitor (NEB) in Biotin Wash Buffer (10 mM Tris HCl pH 7.4, 1 mM EDTA, 100 mM NaCl, 0.05 % Tween-20) for 1 h at 25 °C. The beads were washed by Biotin Wash Buffer and the spun down. Next, 50 μg of the biotinylated total RNAs were diluted in 1 mL Biotin Wash Buffer and incubated with the blocked MyOne C1 beads for 4 h at 4 °C. Beads were washed to remove un-bound RNAs for two times with 1 mL of ChIRP Wash Buffer (2 × SSC, 0.5 % SDS), then washed two time with 1 mL of Biotin Wash Buffer each, followed by two time washes with 1 mL NT2 Buffer (50 mM Tris HCl, pH 7.5, 150 mM NaCl, 1 mM MgCl2, 0.005 % NP-40). All washing was performed at room temperature for 3 min each. The streptavidin beads were then eluted by incubation in 1 mL TRIzol at 25 °C for 10 min and the glycoRNA was extracted and purified by Zymo C column. To separate glycoRNA-L and glycoRNA-S, first the purified glycoRNAs were loaded on low-temperature melting agarose gel and separated by electrophoresis. The gel slices containing glycoRNA-L and glycoRNA-S were collected. 4 volumes of RNA binding buffer were added into the tube containing gel slices and incubated at 50 °C for 10 min. Then added 1 volume of 100 % ethanol and incubated at 50 °C for additional 5 min. The glycoRNA-L and glycoRNA-S were purified by Zymo column as described above.

### 4.11. Western blot

Western blot was performed as described previously [24]. Briefly, Cells quickly rinsed with ice-cold PBS and directly lysed with RIPA buffer (150 mM NaCl), containing proteinase inhibitor cocktail 1 and 3 and phosphatase inhibitor cocktail (Cell Signaling Technology) on ice for 15 min. After centrifugation at 12,000 ×*g* for 15 min at 4 °C, lysates were heated at 95 °C for 10 min in 1× NuPAGE LDS loading buffer (ThermoFisher Scientific) containing 5 mM DTT. Samples were then resolved by SDS-PAGE using AnyKD Criterion TGX Precast Midi Protein Gels (Bio-Rad Laboratories) and transferred to nitrocellulose membranes. Membranes were blocked in blocking buffer and incubated with primary antibodies (diluted in blocking buffer) at 4 °C overnight. After washing three times for 3 min each in 1× PBS with 0.1 % Tween-20 (PBST), membranes were incubated with secondary antibodies at room temperature for 60 min, followed by the same 3× PBST washing. Membranes were finally rinsed in 1× PBS and scanned on iBright 1500 (Invitrogen). Primary antibodies used: mouse monoclonal anti-hSiglec-5 (R&D systems, 1:1000), rabbit anti-VCAM-1 polyclonal antibody (Santa Cruz, 1:1000), rabbit polyclonal anti-β-actin (Santa Cruz, 1:1000). Secondary antibodies used: goat anti-rabbit IgG-HRP (Invitrogen, 1:1000) and rabbit anti-mouse IgG-HRP (Invitrogen, 1:1000).

### 4.12. Cell viability

The cell viability was tested by Trypan Blue Exclusion Test. Briefly, 10^7^ THP1 cells were spun down and then resuspended into 1 ml HBSS buffer and incubated with 0, 1 or 5 μL of RNase cocktail at 37 °C for 10 min. The cells then were mixed with equal volume of 0.4 % Trypan blue (gibco) and counted by a cellometer (Nexcelom Bioscience). The cell viability was presented as all cell numbers-stained cell numbers/all cell numbersX100%.

### 4.13. Statistics

Student’s *t*-test (unpaired, unequal variance) or One-way ANOVA analysis followed by Tukey’s test was used to assess experimental significance.

## Data Availability

Data will be made available on request.
